# Anti-Inflammatory and Neuroprotective Effects of Morin in an MPTP-Induced Parkinson’s Disease Model

**DOI:** 10.3390/ijms231810578

**Published:** 2022-09-12

**Authors:** Dong Geun Hong, Seulah Lee, Jaehoon Kim, Seonguk Yang, Myunggyo Lee, Jinsook Ahn, Haeseung Lee, Seung-Cheol Chang, Nam-Chul Ha, Jaewon Lee

**Affiliations:** 1 Department of Pharmacy, College of Pharmacy, Pusan National University, Busan 46241, Korea; 2 Neurodegenerative Diseases Research Group, Korea Brain Research Institute, Daegu 41062, Korea; 3 Center for Food and Bioconvergence, and Research Institute of Agriculture and Life Sciences, Department of Agricultural Biotechnology, Seoul National University, Seoul 08826, Korea; 4 Department of Cogno-Mechatronics Engineering, College of Nanoscience and Nanotechnology, Pusan National University, Busan 46241, Korea

**Keywords:** anti-inflammation, astrocyte, microglia, morin, neuroprotection, Parkinson’s disease

## Abstract

Neurodegenerative diseases such as Parkinson’s disease (PD) are known to be related to oxidative stress and neuroinflammation, and thus, modulating neuroinflammation offers a possible means of treating PD-associated pathologies. Morin (2′,3,4′,5,7-pentahydroxy flavone) is a flavonol with anti-oxidative and anti-inflammatory effects found in wines, herbs, and fruits. The present study was undertaken to determine whether a morin-containing diet has protective effects in an MPTP-induced mouse model of PD. Mice were fed a control or morin diet for 34 days, and then MPTP (30 mg/kg, i.p.) was administered daily for 5 days to induce a PD-like pathology. We found that dietary morin prevented MPTP-induced motor dysfunction and ameliorated dopaminergic neuronal damage in striatum (STR) and substantia nigra (SN) in our mouse model. Furthermore, MPTP-induced neuroinflammation was significantly reduced in mice fed morin. In vitro studies showed that morin effectively suppressed glial activations in primary microglia and astrocytes, and biochemical analysis and a docking simulation indicated that the anti-inflammatory effects of morin were mediated by blocking the extracellular signal-regulated kinase (ERK)-p65 pathway. These findings suggest that morin effectively inhibits glial activations and has potential use as a functional food ingredient with therapeutic potential for the treatment of PD and other neurodegenerative diseases associated with neuroinflammation.

## 1. Introduction

Parkinson’s disease (PD) is a common age-related neurodegenerative disorder characterized by progressive dopaminergic neuronal cell loss in the substantia nigra (SN), tremors, rigidity, and bradykinesia [1]. In addition to motor symptoms, various nonmotor symptoms such as autonomic nerve dysfunction, sensory cognitive impairment, and sleep disturbance develop [2]. The underlying cause of PD development has not been determined, but some epidemiological studies suggest that exposure to pesticides, herbicides, and heavy metals increases risk [3]. Additionally, though no clear molecular mechanisms have been elucidated, evidence suggests that PD is associated with mitochondrial dysfunction, oxidative stress, and chronic neuroinflammation [4].

Available evidence also indicates that PD is related to the activations of microglia and astrocytes involved in immunity in the striatum (STR) and substantia nigra (SN) of PD patients [5], and it has been reported that resulting neuroinflammation kills dopaminergic neurons and that inflammation is a feature of degenerative neurological diseases such as PD [6]. Thus, inflammation is considered a basic component of neurodegenerative diseases, and microglia are a major causative factor. Accordingly, it has been suggested that the detection of inflammation might enable the early diagnosis of PD and that anti-inflammatories might retard or even prevent the progression of neurodegenerative diseases [7]. Recently, many authors have reported that the modulation of neuroinflammation might have a neuroprotective effect in neurodegenerative diseases [8,9,10]. Polyphenols such as silibinin and baicalein have been reported to elicit neuroprotective effects due to their anti-oxidative and anti-inflammatory properties [11,12]. In addition, recent studies have suggested that the lichen metabolites usnic acid and evernic acid have therapeutic potential in PD due to their anti-inflammatory effects [13,14].

Morin is abundantly expressed in the leaves, stems, branches, and fruits of members of the *Moraceae* family [15]. Various studies have shown that morin has antioxidant and anti-inflammatory effects [16,17]. In addition, its low toxicity levels in vivo indicate chronic morin administration is a practical possibility [18,19]. In our previous study, we showed the anti-inflammatory effects of morin resulted in neuroprotective effects in a mouse model of PD, and that in an acute PD model, intraperitoneal (i.p.) morin administration effectively improved motor dysfunction and prevented dopaminergic neuron loss [20]. However, the potential value of morin as a functional food has not been investigated, particularly in neuroinflammation-related neurodegenerative diseases such as PD. In the present study, we evaluated whether a morin-containing diet could help prevent MPTP-induced PD pathology in mice and studied the mechanism whereby morin modulates neuroinflammatory responses in primary glial cell cultures.

## 2. Results

### 2.1. Body Weights and Metabolic Parameters of Morin-Fed Mice

**Figure 1:**
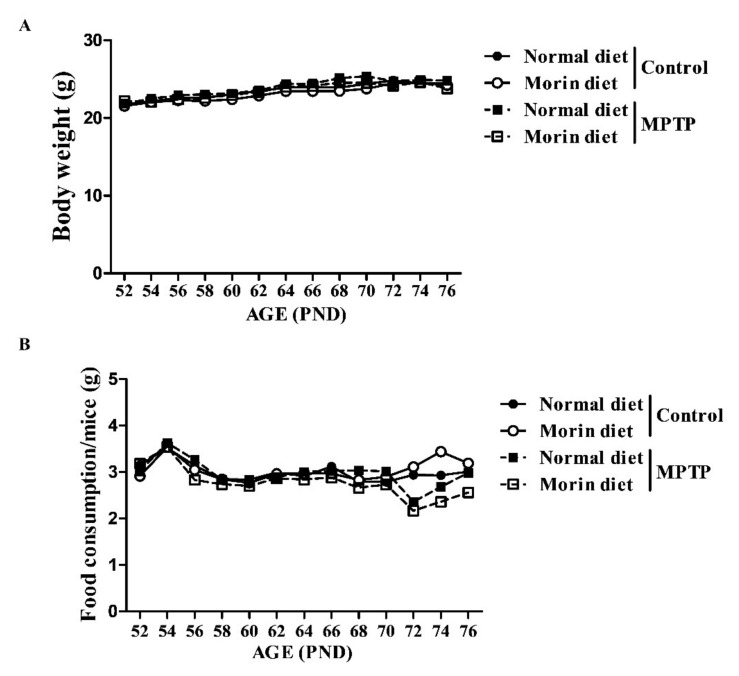
Body weights and food intakes in morin-fed mice. (**A**) Body weights. (**B**) Food intakes. No significant difference in body weight or food intake was observed between the control morin diet group and control normal diet group.

No significant differences in bodyweight or food consumption were observed between the control normal diet group and the control morin diet group (Figure 1A,B). Blood chemistry results for triglyceride (TG), serum non-esterified fatty acids (NEFA), total cholesterol (TC), and glucose were similar in the morin and normal diet control groups (Table 1). These results indicate that dietary intake of morin does not cause side effects such as obesity in vivo.

### 2.2. Morin Effectively Restored Motor Dysfunction in the MPTP-Induced PD Model

**Figure 2:**
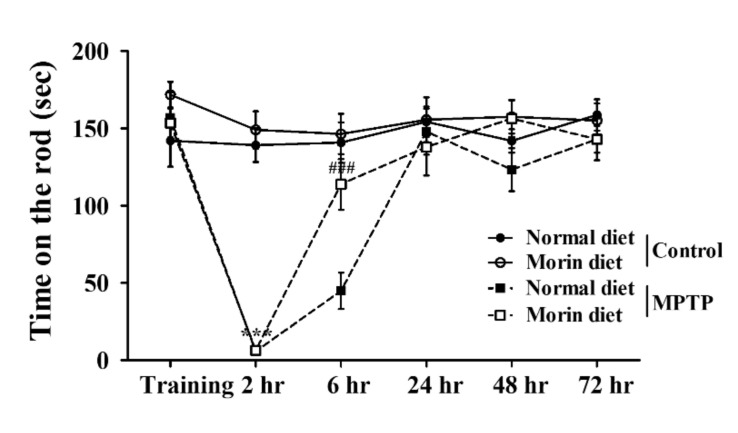
Morin effectively attenuated motor dysfunction in our murine PD Model. Rotarod testing was used to evaluate motor function. Mice were pre-trained for three days to remain on the rod at 15 to 30 rpm. The tests were performed at 2, 6, 24, 48 h, and 72 h after the last MPTP injection at a rod speed of 30 rpm. Data are presented as means ± SEMs. *** *p* < 0.001 vs. control normal diet, ### *p* < 0.001 vs. MPTP normal diet.

We used the rotarod test to investigate the effects of morin in the diet on motor function in our PD model. Mice were pre-trained for 3 days to remain on the rod for 180 s at 30 rpm. Then, MPTP was injected once daily for 5 days from experimental day (ED) 27 to induce a PD-like pathology. Rotarod testing showed the MPTP group exhibited motor dysfunction, and mice in the MPTP morin diet group showed improved motor function at 6 h after last MPTP treatment (Figure 2).

### 2.3. Morin Diet Alleviated MPTP-Induced Dopaminergic Neuronal Losses in STR and SN

**Figure 3:**
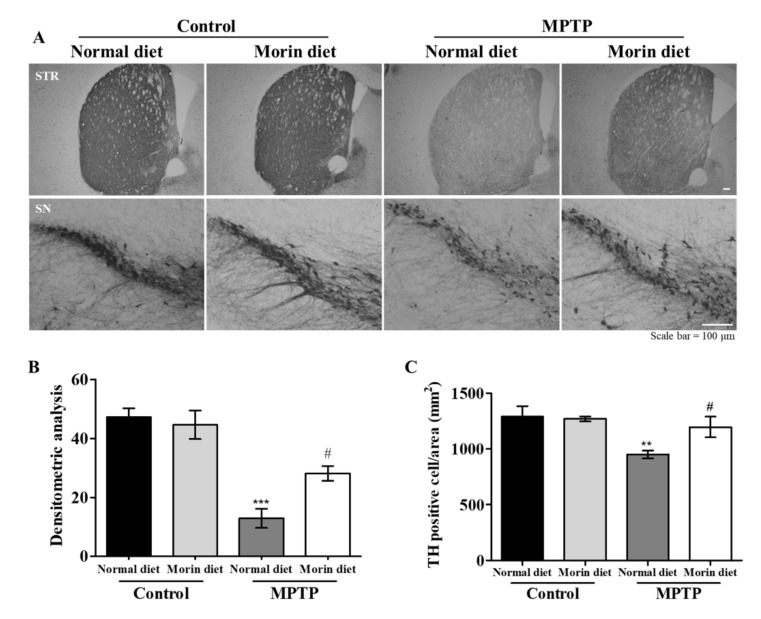
MPTP-induced dopaminergic neuronal losses in STR and SN were alleviated in mice fed the morin diet. (**A**) Immunohistochemistry (IHC) was performed using antibodies against TH (a marker of dopaminergic neurons) in STR. TH levels were lower in the MPTP group than in control group. The morin diet effectively ameliorated MPTP-induced dopaminergic neuronal damage. (**B**) Quantification of TH expressions in STR. Data are presented as means ± SEMs. *** *p* < 0.001 vs. control normal diet, # *p* < 0.01 vs. the MPTP normal diet. (**C**) TH positive dopaminergic neurons were counted in substantia nigra. (*n* = 10–11 mice/group). Data are presented as means ± SEMs. ** *p* < 0.01 vs. control normal diet, # *p* < 0.01 vs. the MPTP normal diet.

The nigrostriatal pathway is a major dopamine pathway that connects STR and SN and is responsible for motor function. To evaluate the neuroprotective effects of morin, we performed immunohistochemistry (IHC) using tyrosine hydroxylase (TH) antibody (a dopamine neuronal marker). TH levels were lower in the MPTP group than in the control group. However, morin in the diet effectively ameliorated MPTP-induced dopaminergic neuronal damage (Figure 3A). For quantitative analysis, TH levels were measured by densitometry in STR (Figure 3B), and TH-positive neurons in SN were counted (Figure 3C). These results showed morin in the diet protected dopaminergic neurons against MPTP-induced neuronal loss/damage.

### 2.4. Mice Fed with the Morin Diet Mitigated Glial Activation in the PD Model

**Figure 4:**
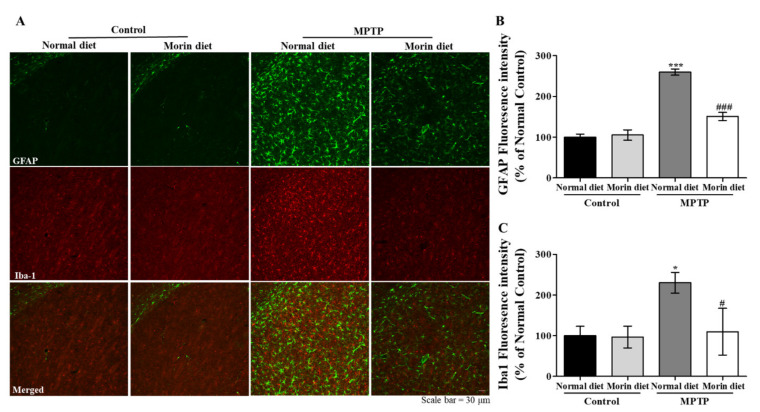
Morin attenuated glial activation in our PD model. Double immunohistochemistry was performed using antibodies against GFAP (an astrocyte marker) and Iba-1 (a microglial marker) to evaluate neuroinflammation. Increased astroglial activations were observed in the MPTP normal diet, but these activations were suppressed by dietary morin. (**A**) Striatum. (**B**) Quantitative analysis of GFAP (green) fluorescence intensities. Data are expressed as means ± SEMs. *** *p* < 0.001 vs. control normal diet, ### *p* < 0.001 vs. MPTP normal diet. (**C**) Quantitative analysis of Iba-1 (red) fluorescence intensities. Data are expressed as means ± SEMs. * *p* < 0.05 vs. control normal diet, # *p* < 0.05 vs. MPTP normal diet.

Double immunohistochemistry was performed using antibodies to glial fibrillary acidic protein (GFAP) (astrocyte marker) and Iba-1 (microglial marker) to evaluate neuroinflammation. Increased astrocyte and microglia activations were observed in the MPTP normal diet group, but less astrocyte and microglia activations were observed in the MPTP morin diet than in the MPTP normal diet group (Figure 4A). Bar graphs of fluorescence intensity also showed that the morin diet significantly inhibited MPTP-induced astrocyte and microglia activation (Figure 4B,C). These results showed that the anti-inflammatory effects of the morin diet were associated with its neuroprotective effects.

### 2.5. Morin Inhibited MPP^+^-Induced Astrocyte Activation

**Figure 5:**
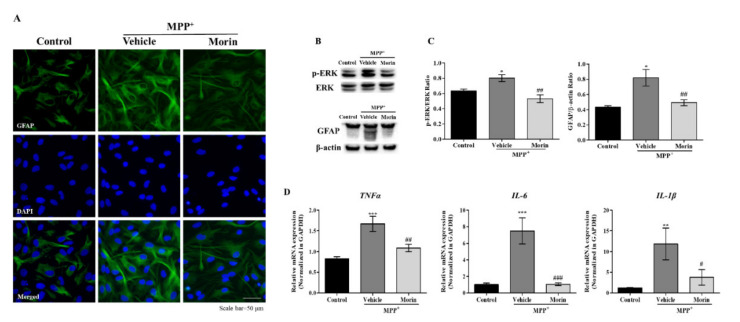
Morin effectively suppressed the effects of glial activation on astrocytes. (**A**) Representative images showing that morin diminished GFAP fluorescence intensity (an astrocyte marker). Cells were counterstained with DAPI. (**B**) Western blot showed that morin repressed MPP⁺-induced ERK phosphorylation and glial activation in primary astrocytes (*n* = 3). (**C**) Quantitative densitometric analysis of Western blots. β-Actin was used as a loading control. Data are presented as means ± SEMs. * *p* < 0.05 vs. control, ## *p* < 0.01 vs. vehicle (**D**). Real time-PCR showed that morin significantly reduced MPP+-induced inflammatory cytokines level increases. Data are presented as means ± SEMs. *** *p* < 0.001 and ** *p* < 0.01 vs. control, ### *p* < 0.001, ## *p* < 0.01 and # *p* < 0.05 vs. vehicle.

To determine whether morin had a beneficial anti-inflammatory effect, primary astrocytes were pretreated with morin for 6 h and then co-treated for 24 h with 1-methyl-4-phenylpyridinium (MPP^+^), which is known to trigger A1-type astrocyte activation [10,13]. Eastern blot and ICC showed pretreatment with 100 μM morin significantly attenuated MPP^+^-induced ERK phosphorylation and GFAP expression (Figure 5A–C), and real-time PCR showed morin significantly inhibited MPP^+^-induced inflammatory cytokine production in astrocytes (Figure 5D). These results suggest morin regulates neuroinflammation by inhibiting ERK-mediated inflammatory astrocyte activation by MPP^+^.

### 2.6. Morin Effectively Reduced Microglial Activation

**Figure 6:**
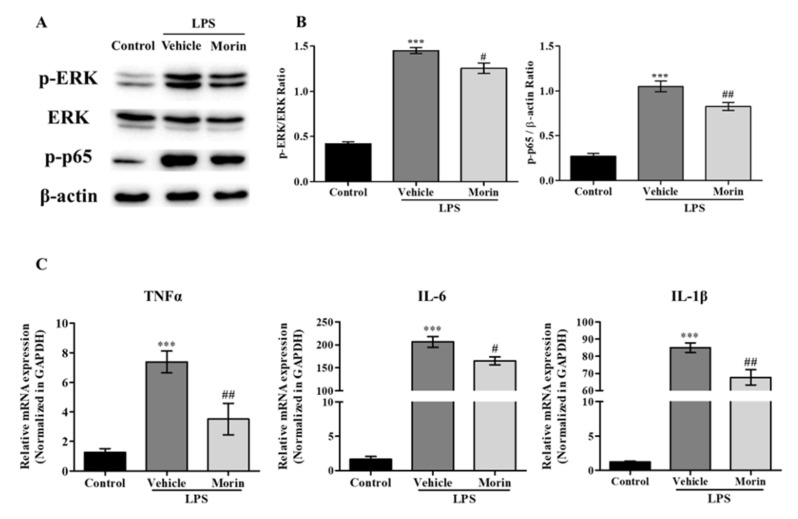
Morin effectively suppressed glial activations on microglia. (**A**) Western blot analysis showed that morin inhibited the LPS-induced phosphorylations of ERK and p65 in primary microglia (*n* = 3). (**B**) Quantitative densitometric analysis of Western blots. β-Actin was used as a loading control. Data are presented as means ± SEMs. *** *p* < 0.001 vs. control, ## *p* < 0.01 and # *p* < 0.05 vs. vehicle. (**C**) Real time-PCR showed that morin significantly reduced LPS-induced increases in inflammatory cytokines levels. Data are presented as means ± SEMs. *** *p* < 0.001 vs. control, ## *p* < 0.01 and # *p* < 0.05 vs. vehicle.

The anti-inflammatory effects of morin were also investigated in microglia. Primary cultured microglia were pretreated with morin for 6 h and then co-treated with lipopolysaccharide (LPS) for 30 min. Western blot showed that LPS increased the phosphorylations of p65 and ERK in primary microglia and that morin pretreatment significantly reduced this effect (Figure 6A,B). In addition, real-time PCR showed that morin significantly inhibited LPS-induced inflammatory cytokine increases in primary microglia (Figure 6C). These results suggest that morin exerts its anti-inflammatory effects in LPS-stimulated microglia activation by suppressing the ERK-p65 pathway.

### 2.7. Docking Simulation Analysis and the Binding Affinity of Morin for ERK

**Figure 7:**
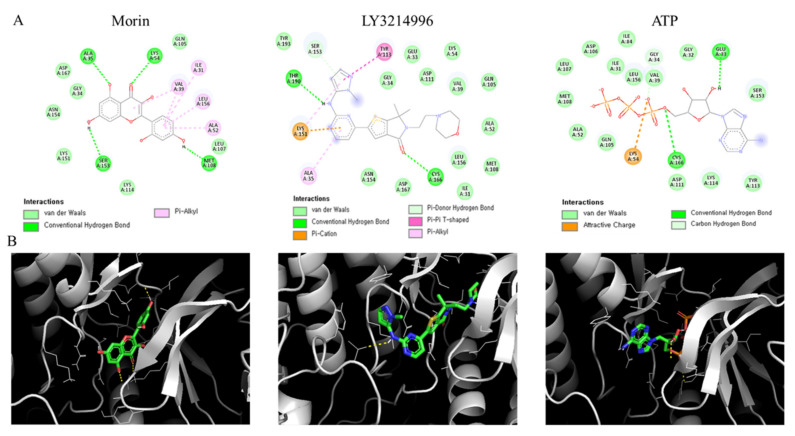
In silico molecular docking analysis showed binding between ERK2 and morin, LY3214996, or ATP. (**A**) 2D diagrams showing the types of contacts formed between ERK2 and the indicated ligands. The colors of dotted lines represent types of interactions between ERK2 binding site residues and the indicated ligands. (**B**) 3D interactions showing the binding pocket structure of ERK2 and the indicated ligands.

Previous biochemical analysis showed that morin mitigated glial activations in astrocytes and microglia by blocking the ERK-p65 pathway. Docking simulations were performed to determine whether morin interacts directly with ERK using the well-known ERK inhibitor LY3214996 as a positive control. We found morin and LY3214996 bind to the ATP-binding site of ERK. The hydroxyl group of morin and Ala, Lys, Met, and Ser of the active site formed hydrogen bonds, and the dihydroxyl phenyl ring of morin formed hydrophobic interactions with Ile, Val, Ala, and Leu (Figure 7A,B). The binding affinities of ERK with morin, ATP, and LY3214996 were −8.0, −6.5, and −8.2 kcal/mol, respectively (Table 2). Docking simulations showed that morin and LY3214996 bound to ERK with similar affinities and that these affinities were greater than that of ATP.

## 3. Discussion

Parkinson’s disease is a neurodegenerative disease that mainly affects the elderly, and recent studies indicate that neuritis caused by the activations of microglia and astrocytes may cause Parkinson’s disease [21]. Glial cells are essential for regulating normal brain homeostasis, but when dysfunctional contribute to neurodegenerative brain disease [22]. Uncontrolled glial cells release cytokines, causing chronic neuritis, which can cause neurodegenerative diseases, and targeting this cytokine release using anti-inflammatories can delay or prevent the onset of neurodegenerative diseases [23,24].

Morin has been reported to inhibit ROS generation and NF-κB-mediated inflammatory responses [20], and the anti-inflammatory effects of morin observed in the present study suggest that morin may be a useful dietary supplement for those with Parkinson’s disease. In the present study, morin in the diet was found to restore MPTP-induced motor dysfunction in a mouse PD model and inhibit MPTP-induced loss of dopaminergic neurons and glial cell activation. In a previous study, we attributed the neuroprotective effects of morin to its anti-inflammatory effects in a mouse model of PD [20]. Plants and herb extracts, including morin extracts, have long been used to treat various diseases [15,25], and nutraceuticals and herbal medicines are now viewed as important aspects of human health care because of their low toxicities, availabilities, and fewer side effects than synthetic drugs [15]. However, because morin has poor water solubility and low bioavailability, it must be combined with other substances [26]. Therefore, in this study, a feed containing morin was prepared and we investigated whether morin in the diet had the same efficacy as morin administered. We found morin-induced anti-inflammatory effects manifested as neuroprotective effects in our murine model PD, and no side effects were observed.

Serum analysis was performed to determine whether morin had side effects. TG is an important clinical feature of metabolic syndrome, and high serum NEFA levels are associated with the risks of abdominal obesity and cardiovascular disease [27,28]. However, when we compared blood test results, no difference was found between the morin and treatment-naïve control groups. On the other hand, TG, NEFA, and glucose levels were lower in the MPTP control group, which we believe was caused by reduced food consumption due to the PD-like pathology induced by MPTP. Thus, we concluded that morin in the diet has no perceivable effect on metabolism.

A considerable amount of evidence indicates that various polyphenols have neuroprotective effects in many neurodegenerative diseases due to their anti-inflammatory and antioxidant effects [11,12,29]. Moreover, it has been widely reported that antioxidants can suppress neuroinflammatory responses [30,31]. However, in a previous study, we found that antioxidants alone did not prevent the activation of inflammatory astrocytes [31], which indicates that the anti-inflammatory effects of morin in primary astrocytes are not mediated by its antioxidative activity. In the current study, we evaluated the anti-inflammatory effects of morin in two different glial activation models, and interestingly, we found that ERK plays an important role in both glial activation-signaling mechanisms. ERK is a protein-serine/threonine kinase that participates in the Ras-Raf-MEK-ERK signaling cascade, which regulates various processes, including cell survival, differentiation, metabolism, and transcription [32]. However, chronic activation of microglia can induce neuronal damage due to the release of molecules like inflammatory cytokines [33]. In PD, microglia are activated by toxins to attack adjacent dopaminergic neurons, and α-synuclein activates the p38, ERK, and JNK pathways to produce IL-1β and TNF-α and promote inflammation [34]. ERK and NF-κB are key regulators of inflammatory response, and LPS and MPP+ increase each activated microglia and astrocytes [10], which in turn, activate NF-κB (a key proinflammatory transcription factor) and release proinflammatory cytokines such as TNFα, IL-6, and IL-1β [35]. The upregulation of phosphorylated ERK has been reported in animal models of PD, and oxidative stress has been shown to enhance ERK phosphorylation [31]. Various studies have shown that the inhibition of ERK phosphorylation and activation can have anti-Parkinsonian effects [36,37]. The present study shows that morin effectively inhibited the phosphorylations of ERK and p65 in activated astrocytes and microglia and attenuated cytokines levels. These results suggest that morin has neuroprotective effects and that these are due to ERK-specific mediated anti-inflammatory effects and its antioxidant effects.

We subsequently investigated how morin inhibits ERK by molecular docking analysis (Figure 7A,B). Morin and LY3214996 had the highest affinity for ERK, while ATP displayed relatively low affinity. Ala, Lys, Met, and Ser in the active site of ERK formed hydrogen bonds with the hydroxyl groups of morin, and Ile, Val, Ala, and Leu interacted hydrophobically with the dihydroxy phenyl ring of morin. Although LY3214996 binds to the same site as morin and ATP, we found that another well-known inhibitor, BVD-523, binds to a different site with an affinity of −7.8 kcal/mol (data not shown) [38]. Our docking analysis showed that morin prefers to bind to the ATP-binding site of kinases, which concurs with a previous report that morin directly binds to the ATP binding site of GSK3β [39].

In summary, we found that morin in the diet effectively prevented the development of a PD-like pathology and exhibited anti-inflammatory effects in a mouse model of PD. Furthermore, our in vitro studies showed that morin inhibited astrocyte and microglial activations by downregulating the ERK-p65 pathway. Therefore, the present study suggests that morin has potential use as a dietary supplement with a protective effect against PD.

## 4. Methods

### 4.1. Morin Mouse Feed

The morin diet was provided by the Center for Food and Bioconvergence Institute at Seoul National University. The normal diet was composed of 52% starch and sugars, 21% amino acids, and 5% fat, and the morin diet was prepared by adding 1% morin hydrate to the normal diet. Metabolizable energies of the normal diet for carbohydrate, protein, and fat were 8.7, 3.6, and 1.9 kJ/g, respectively, as estimated using Atwater energy equivalents. Further, details of the diet are provided in the Table 3.

### 4.2. Animals and Diet Administration

Six-week-old male C57BL/6N mice (weighing 20–25 g) were purchased from DaehanBiolink (Chungbuk, Korea). Mice were randomly housed at five animals per cage, maintained under controlled conditions (20–23 °C, 12 h light-dark cycle), and fed the morin diet or the normal diet for 4 weeks. Animals were administered MPTP intraperitoneally (i.p.) at 30 mg/kg daily for 5 days in the MPTP group (both *n* = 10–11)), or 5% ethanol in phosphate-buffered saline (PBS) containing 2% Tween-20 in the control group (both *n* = 10). Mice were fed the morin diet until the end of the experiment. The animal protocol used was reviewed and approved by the Animal Care Committee of Pusan National University Institutional Animal Care Committee (PNU-IACUC, approval number PNU-2021-2961).

### 4.3. Motor Performance Testing

MPTP-induced motor dysfunction was evaluated using a rotarod test, as described previously [10,13]. All mice underwent training trials for three days to ensure they could maintain themselves on the rod for 180 s at a rod speed of 30 rpm. Training sessions were performed using four consecutive runs. All mice were tested using a rotarod speed of 30 rpm for 180 s at 2, 6, 24, 48, and 72 h after final MPTP administration.

### 4.4. Biochemical Analysis of Serum

Blood samples were collected from animals in each group after sacrifice on ED 34. Serum samples were prepared by centrifugation at 3000 rpm for 30 min at 4 °C. Serum triglyceride (TG), non-esterified fatty acid (NEFA), total cholesterol (TC), and glucose levels were analyzed using kits purchased from Bioassay Systems (Hayward, CA, USA).

### 4.5. Tissue Preparation 

For histologic analysis, mice were anesthetized 72 h after last MPTP administration (on ED73) with ethyl ether and perfused intracardially with 0.1 M PBS (pH 7.4) containing 0.9% NaCl and then with 0.1 M PBS containing 4% paraformaldehyde. The brains were removed, post-fixed, and cryoprotected. The STR and SN were delineated from 1.54 to −0.34 mm and −2.54 to −3.88 mm from bregma, according to the Paxinos mouse brain atlas. Cryoprotected brains were sectioned serially at 40 μm in the coronal plane using a freezing microtome (MICROM, Walldorf, Germany), and then stored at 4 °C in Dulbecco’s phosphate-buffered saline (DPBS) solution containing 0.1% sodium azide.

### 4.6. Diaminobenzidine Immunohistochemistry

Brain sections (40 µm) were incubated with 0.6% H2O2 in Tris-buffered saline (TBS; pH 7.5) for 30 min, washed with TBS (3 × 10 min), treated with TBS/0.1% Triton X-100/3% goat serum (TBS-TS) for 30 min, and incubated with the primary antibody (anti-tyrosine hydroxylase (TH) antibody in TBS-TS at 4 °C overnight. Sections were then incubated at room temperature with the appropriate biotinylated secondary goat anti-mouse and anti-rabbit IgG antibodies in TBS for 3 h, and then in an avidin-biotin complex (ABC) solution (Vectastain ABC reagent Elite Kit, Vector Laboratories, Burlingame, CA, USA) in TBS at room temperature for 1 h. After development in diaminobenzidine (DAB) solution, images were obtained using a Nikon ECLIPSE TE 2000-U microscope (Nikon, Tokyo, Japan). The quantitative results for dopaminergic neurons were obtained as follows. Five to six sections per mouse containing the SN were taken, and the numbers of TH-positive dopaminergic neurons in sections were counted and then divided by section areas. The averages of TH-positive neurons in five to six sections per mouse were calculated. To quantitatively analyze TH immunostaining in STR, TH expression intensities in STR (five to six sections per mouse) were measured using a FluorChem SP software (Alpha Innotech, San Leandro, CA, USA).

### 4.7. Double Fluorescence Immunohistochemistry

Brain sections (40 µm) were blocked with TBS-TS for 30 min at room temperature and incubated with primary antibodies, i.e., anti-glial fibrillary acidic protein (GFAP) antibody (mouse polyclonal; 1:500, Cell Signaling, MA, USA) and anti-ionized calcium-binding protein (Iba-1) antibody (rabbit polyclonal; 1:500, Wako, Tokyo, Japan) in TBS-TS overnight at 4 °C. Sections were then washed with TBS, incubated with anti-mouse IgG labeled with Alexa Fluor 488 (3 μL/mL) and anti-rabbit IgG labeled with Alexa Flour 568 (3 μL/mL) for 3 h at room temperature. Images were obtained using a ZEISS LSM800 confocal microscope (Oberkochen, Germany). Two or three sections per mouse containing SN or STR were taken, and fluorescence intensities over full images in left and right hemispheres were measured using Image J software, and averaged.

### 4.8. Primary Glial Cell Culture

Primary astrocyte cultures were established using cells from the cortices of Sprague Dawley (SD) rat pups at postnatal day 1 or 2 (DaehanBiolink Co., Ltd., Chungbuk, Korea). Briefly, cortices were dissected and diffused in ice-cold Hanks’ balanced salt solution (HBSS; Welgene, Daegu, Korea). Cells were treated with 0.25% trypsin for 15 min at room temperature, washed with HBSS, mechanically dissociated, and plated in Dulbecco’s modified Eagle’s medium (nutrient mixture F12 (DMEM/F12), medium (Gibco, Grand Island, NY, USA) containing 10% FBS (Welgene, Daegu, Korea) and 1% penicillin–streptomycin (Welgene, Daegu, Korea) on poly-L-lysine-coated (Sigma-Aldrich, St. Louis, MO, USA) plastic culture dishes. Experiments were performed using 14 to 21 day cultures.

### 4.9. Immunocytochemistry 

Primary glial cells were seeded in 35 mm poly-L-lysine-coated plastic culture dishes and pretreated with morin (100 μM in DMEM medium containing 0.1% DMSO) for 6 h. Cells were then co-treated with MPP⁺ (250 μM) or LPS (1 μg/mL) for 24 h, washed with PBS, fixed with 4% paraformaldehyde (PFA) in PBS (pH 7.4) for 15 min at 37 °C, blocked with tris-buffered saline and then with Triton X-100 and goat serum (TBS-TS) for 30 min, and incubated with GFAP antibody (mouse monoclonal; 1:1000, Cell Signaling Technology, Danvers, MA, USA) or Iba1 antibody (rabbit polyclonal; 1:500, Wako, Tokyo, Japan) overnight at 4 °C. The following day, cells were washed, incubated with anti-mouse IgG labeled with Alexa Fluor 488 (3 μL/mL) or anti-rabbit IgG labeled with Alexa Flour 568 (3 μL/mL) for 3 h at room temperature, and then incubated in DAPI solution (1 μg/mL) for 30 min at 37 °C.

### 4.10. Western Blot Analysis

Samples were loaded on 10% SDS-polyacrylamide gels for Western blot analysis and then transferred to Immobilon PSQ transfer membranes (Millipore, Bedford, MA, USA). Membranes were immediately incubated in TBS-T (Tris-HCl-based buffer containing 0.2% Tween 20, pH 7.5) containing 5% nonfat milk for 30 min at room temperature, washed, and incubated with primary antibodies: GFAP (mouse; 1:1000; Abcam, Cambridge, MA, USA), phospho-extracellular signal-regulated kinase (p-ERK) (rabbit; 1:1000, Cell Signaling Technology, Danvers, MA, USA), ERK (rabbit; 1:1000 Cell Signaling, MA, USA), p-p65 (rabbit monoclonal; 1:500, Cell Signaling MA, USA), and β-actin (mouse monoclonal; 1:10,000; Sigma-Aldrich) in TBS-T overnight at 4 °C. The membranes were then washed for 10 min and incubated with secondary monoclonal anti-mouse and anti-rabbit antibody conjugated with horseradish peroxidase (1:10,000; Santa Cruz Biotechnology, Santa Cruz, CA, USA) in TBS-T buffer for 2 h at room temperature. Membranes were developed by enhanced ECL and photographed using a cooled CCD camera system (ATTO Ez-Capture; Atto Corp., Tokyo, Japan). Fold changes in relative protein levels were quantified by densitometry using either total MAPK (ERK) or β-actin as loading controls. 

### 4.11. RNA Isolation and Real-Time Polymerase Chain Reaction (Real-Time PCR)

Cells were homogenized with Trizol reagent (Invitrogen; Carlsbad, CA, USA) and shaken vigorously with chloroform for 15 min. Aqueous phases were then transferred to fresh tubes, isopropanol was added, incubated for 15 min at 4 °C, and centrifuged for 15 min at 12,000 rpm. After removing supernatants, pellets were washed with 75% ethanol and centrifuged for 5 min at 8000 rpm. The RNA pellets obtained were dried and dissolved in diethylpyrocarbonate water, and mRNA concentrations were calculated. mRNA was reverse transcribed to cDNA using SuPrimeCript RT Premix (Genetbio Inc.; Daejeon, Korea), and real-time PCR was performed using SYBR green master mix (BIOLINE; Taunton, MA, USA) and the CFX Connect System (Bio-Rad Inc.; Hercules, CA, USA). The primer sequences used were as follows: tumor necrosis factor-α (TNF-α) (NM_012675.3) (5′-ATT GCT CTG TGA GGC GAC TG-3′ and 5′-GGG GCT CTG AGG AGT AGA CG-3′); interleukin (IL)-6 (NM_012589.2) (5′-TCA TTC TGT CTC GAG CCC AC-3′ and 5′-GAA GTA GGG AAG GCA GTG GC-3′); IL-1β (NM_031512.2) (5′-AAA ATG CCT CGT GCT GTC TG-3′ and 5′-CCA CAG GGA TTT TGT CGT TG-3′); GAPDH (NM_017008.4) (5′-AGA CAG CCC CAT CTT GT-3′ and 5′-ACG GTG AGT CTT CTG ACA CC-3′).

### 4.12. Molecular Docking Analysis

The reported three-dimensional (3D) protein structure of ERK2 was downloaded from AlphaFold Protein Structure Database (https://alphafold.ebi.ac.uk/ (accessed on 17 January 2022), AF-P28482-F1-model_v2) in Protein Data Bank (PDB) File format. The 3D structures of the three ligands: Morin, CID: Z5281670; LY3214996, CID: 121408882; ATP, CID: 5957; were obtained from the PubChem database (https://pubchem.ncbi.nlm.nih.gov/, accessed on 17 January 2022) in Structure Data File (SDF) format and converted to PDB file format using AutoDockFRprepare_ligand software [PMID: 26629955]. Computational docking simulations were conducted between ERK2 protein and morin, LY3214996, or ATP using AutoDock Vina 1.2. in AMDock version 1.5.2 in ‘Simple Docking’ mode [PMID: 32938494]. The grid box was set to 70, 70, and 70 Å for x, y, and z, respectively, and centered on the following xyz-coordinates to explore the entire predicted binding site: Morin, 1.10, −9.10, and −11.10 Å; LY3214996, 0.70, −10.50, and 4.90 Å; ATP, 0.20, −6.20, and −13.70 Å. Results of the docking conformation and receptor–ligand interactions were visualized using Discovery Studio Visualizer v21.1.0.20298 (BIOVIA, San Diego, CA, USA).

### 4.13. Statistical Analysis

Analysis of variance (ANOVA) and t-test were used to determine the significance of differences between groups, and post hoc tests were performed to compare groups using Tukey’s multiple comparisons. The analysis was performed using Prism ver. 7.0. (GraphPad Software Inc., San Diego, CA, USA), and *p* values < 0.05 were considered significant.

## Figures and Tables

**Table 1:** Metabolic parameters of morin-fed mice. Data are represented as means ± SEMs. ** *p* < 0.01 and * *p* < 0.05 vs. control morin diet group, # *p* < 0.05 vs. MPTP normal diet group.

	Control		MPTP	
	Normal Diet	Morin Diet	Normal Diet	Morin Diet
Body weight (g)	24.52 ± 0.3	24.24 ± 0.4	24.85 ± 0.4	23.82 ± 0.3
Triglyceride (mg/dl)	85.4 ± 16.2	76.2 ± 5.9	51 ± 8.8	30.6 ± 6.5 *
NEFA (mg/dl)	814 ± 127.1	776 ± 127.4	444 ± 46	440 ± 111.7
Glucose (mg/dl)	284.5 ± 11.4	283.2 ± 23.2	257.8 ± 13.2	211.2 ± 6.1**#
Total Cholesterol (mg/dl)	75.4 ± 10.5	96.6 ± 3.6	96.3 ± 3.3	93.1 ± 2.8

**Table 2:** Binding affinities and binding residues as determined by molecular docking.

Compounds	Binding Affinity (kcal/mol)	Binding Residues
Morin	-8.0	MET108, ALA52, LEU156, ILE31, VAL39, LYS54, ALA35, SER153
ATP	-6.5	GLU33, CYS166, GLY34, LYS54
LY3214996	-8.2	CYS166, ALA35, LYS151, THR190, SER153, TYR113

**Table 3:** Composition of the normal diet and energy contributions of ingredients.

Nutritional Composition		
Nitrogen Free extract of starch and sugars		52.0%
Crude protein		21.4%
Crude at		5.1%
Crude ash		5.4%
Crude fiber		4.0%
Moisture		12.1%
	KJ/g	Kcal/kg
Metabolizable energy	14.2	3395
Energy from proteins	3.6	856
Energy from lipids	1.9	459
Energy from starch and sugars	8.7	2080

## Data Availability

The data that support the findings of this study are available from the corresponding author upon reasonable request.
